# Gluten-free diet in the management of patients with irritable bowel syndrome, fibromyalgia and lymphocytic enteritis

**DOI:** 10.1186/s13075-014-0505-1

**Published:** 2014-12-23

**Authors:** Umberto Volta

**Affiliations:** Department of Medical and Surgical Sciences, University of Bologna, via Massarenti 9, Bologna, 40138 Italy

## Abstract

An evaluation of the effect of 1 year of a gluten-free diet was performed in patients with irritable bowel syndrome and fibromyalgia syndrome displaying lymphocytic enteritis. Gluten withdrawal produced a slight but significant improvement of the functional symptoms, suggesting that gluten might be partly responsible for this clinical picture. This hypothesis should be confirmed by a double-blind placebo-controlled trial since it cannot be ruled out that the studied patients displayed a subjective sensation of improvement due to the placebo effect of gluten withdrawal. Further investigations are needed before recommending gluten withdrawal in patients with fibromyalgia and lymphocytic enteritis.

In their paper published in a recent issue of *Arthritis Research and Therapy*, Rodrigo and colleagues evaluated the effect of 1 year of a gluten-free diet on the clinical evolution of irritable bowel syndrome (IBS) plus fibromyalgia syndrome (FMS) in patients with lymphocytic enteritis (LE) [1]. The study sample included 97 adult females with IBS and FMS, of whom 58 had LE and the remaining 39 had a normal intraepithelial lymphocytic (IEL) count. All subjects fulfilled the Rome III criteria for IBS and the American College of Rheumatology 1990 criteria for FMS and none of them satisfied the diagnostic criteria for celiac disease diagnosis (absence of villous atrophy and negativity for tissue transglutaminase antibodies).

IBS and FMS are two chronic functional disorders that are found in a high number of people in the general population and are frequently detected in the same subject [2]. A subset of patients complaining of IBS and FMS displays LE, a morphological finding that by itself is not specific for celiac disease, also being found in many other pathological conditions such as food allergy, autoimmune disorders, *Helicobacter pylori* infection, nonsteroidal anti-inflammatory drug treatment and common variable immunodeficiency [3].

The spectrum of gluten-related disorders has recently acquired a new syndrome, defined as nonceliac gluten sensitivity according to the criteria established in the two Consensus Conferences held in London and Munich [4]. This new clinical entity is characterized by IBS-like symptoms and several extraintestinal manifestations occurring after gluten ingestion in patients without celiac disease and wheat allergy. In a recent prospective multicenter survey of 486 patients with nonceliac gluten sensitivity, IBS and FMS were respectively detected in 47% and 31% of cases and about one-third of these patients had LE [5].

Along with IBS-related and FMS-related symptoms, the patients studied by Rodrigo and colleagues also showed other manifestations resembling the clinical picture of nonceliac gluten sensitivity such as skin rash, cognitive dysfunction, headache, numbness, anxiety and depression [1].

In Rodrigo and colleagues’ paper, the gluten-free diet produced a slight but significant improvement of both IBS-related (chronic abdominal pain, changes in intestinal habit, bloating) and FMS-related symptoms (chronic widespread pain, generalized tender points, fatigue and restless sleep) in the LE subgroup versus the non-LE subgroup. These results stress the potential role of gluten as a trigger of the clinical manifestations of IBS and FMS and indicate that LE might be useful to identify those patients who potentially benefit from gluten withdrawal. One relevant limitation of this study is the lack of a double-blind placebo-controlled challenge, which is the only procedure to confirm the role of gluten proteins in the development of these clinical manifestations. Indeed, it cannot be ruled out that some patients displayed a subjective sensation of improvement due to the placebo effect of a gluten-free diet [6]. The search for antigliadin antibodies could be of help to elucidate whether gluten can be partly responsible for the clinical picture observed in Rodrigo and colleagues’ patients. Indeed, antigliadin antibodies (particularly those belonging to the IgG class) are the only marker observed in patients with symptoms elicited by gluten ingestion, being positive in more than 50% of cases [7]. These antibodies are not specific for gluten-related symptoms, but their finding in patients with symptoms potentially evoked by gluten ingestion should be regarded as an indication for a gluten-free diet trial in patients with LE [8]. Antigliadin antibodies of the IgG class are closely related to the gluten-induced symptoms and tend to disappear very quickly (within a few weeks) together with the remission of symptoms after a gluten-free diet [9].

An interesting finding emerging from the Spanish study is that about 20% of IBS/FMS patients with LE had relatives with celiac disease, whereas no familial case of celiac disease was observed among patients without LE [1]. In the same guise, familial cases of FMS were found, although to a lesser extent, only in the group with LE (7%). These data suggest that first-degree relatives of IBS/FMS patients with LE should be carefully investigated for the possible presence of undetected cases of celiac disease and FMS. For LE, the mean IEL number reported in Rodrigo and colleagues’ paper was 35/100. This result confirms that LE found in gluten-sensitive patients is mild, with a lower mean IEL number than that usually observed in celiac disease patients (usually >40/100) [10].

The caution in the conclusions of Rodrigo and colleagues’ study is appreciable and shareable. A gluten-free diet is not appropriate in patients with IBS/FMS with normal intestinal mucosa (normal IEL count). Moreover, although the reported results suggest a significant improvement of symptomatology after a gluten-free diet in the LE subgroup, further studies including double-blind placebo-controlled trials are needed before proposing gluten withdrawal in IBS/FMS patients with LE.

## Abbreviations

FMS: Fibromyalgia syndrome
IBS: Irritable bowel syndrome
IEL: Intraepithelial lymphocytic
LE: Lymphocytic enteritis
